# The developmental basis of mesenchymal stem/stromal cells (MSCs)

**DOI:** 10.1186/s12861-015-0094-5

**Published:** 2015-11-20

**Authors:** Guojun Sheng

**Affiliations:** Sheng Laboratory, International Research Center for Medical Sciences, Kumamoto University, Kumamoto, 860-0811 Japan

## Abstract

**Background:**

Mesenchymal Stem/Stromal Cells (MSCs) define a population of progenitor cells capable of giving rises to at least three mesodermal lineages in vitro, the chondrocytes, osteoblasts and adipocytes. The validity of MSCs in vivo has been questioned because their existence, either as a homogeneous progenitor cell population or as a stem cell lineage, has been difficult to prove. The wide use of primary MSCs in regenerative and therapeutic applications raises ethical and regulatory concerns in many countries. In contrast to hematopoietic stem cells, a parallel concept which carries an embryological emphasis from its outset, MSCs have attracted little interest among developmental biologists and the embryological basis for their existence, or lack thereof, has not been carefully evaluated.

**Methods:**

This article provides a brief, embryological overview of these three mesoderm cell lineages and offers a framework of ontological rationales for the potential existence of MSCs in vivo.

**Results:**

Emphasis is given to the common somatic lateral plate mesoderm origin of the majority of body’s adipose and skeletal tissues and of the major sources used for MSC derivation clinically. Support for the MSC hypothesis also comes from a large body of molecular and lineage analysis data in vivo.

**Conclusions:**

It is concluded that despite the lack of a definitive proof, the MSC concept has a firm embryological basis and that advances in MSC research can be facilitated by achieving a better integration with developmental biology.

## Background

The concept of mesenchymal stem/stromal cells (MSCs) [1, 2], also referred to as skeletal stem cells [3] or adipose stem cells [4], was first introduced by Alexander Friedenstein about half a century ago. Building on the hematopoietic stem cell (HSC) work pioneered by another Russian scientist Alexander Maximow, Friedenstein described a population of bone marrow derived cells which are distinct from the HSC population and are osteogenic in vivo and clonogenic in vitro [5–7]. These bone marrow-derived MSCs were later shown to be able to self-renew, form colonies and differentiate into a multitude of mesodermal cell types in vitro [8]. MSC populations with similar multi-lineage differentiation potentials in vitro have since been obtained from many non-bone marrow tissues [9], including the adipose tissue [10, 11], amniotic fluid [12, 13], placenta [14], umbilical cord [15–17] and peripheral blood [18]. Clinical relevance of MSCs has been highlighted by their capacity for in vivo differentiation and engraftment and by their efficacy in promoting wound healing, tissue regeneration and immunosuppression [19–25].

Common for a field attracting a wide scope of interest from researchers, MSC biology has witnessed confusions and controversies concerning its name, definition, isolation and characterization criteria, in vivo relevance, and institutional and ethical regulations of its clinical use. In an attempt to standardize studies in this field, the International Society for Cellular Therapy came up with guidelines in 2006 for MSC characterization [1]. The name “multipotent mesenchymal stromal cells” was preferred and three minimal criteria were outlined: 1) being plastic-adherent in culture; 2) exhibiting a set combination of surface antigens (CD73+, CD90+, CD105+, CD34-, CD45-, CD11b-, CD14-, CD19-, CD79a- and HLA-DR-); and 3) being able to differentiate in vitro into osteoblasts, chondrocytes and adipocytes. These standards, however, have not been widely adopted and criteria for MSC isolation and identification continue to vary, making cross-study comparison difficult [3, 26–31]. As a consequence, physiological nature of their therapeutic effect and cellular and molecular nature of their differentiation potentials in vivo remain ill-characterized.

This article will take an embryological approach to evaluate the evidence for the possible existence of MSCs in vivo. From the perspectives of both cell lineage specification and mesoderm germ layer patterning, developmental ontogeny of the three main mesoderm cell types of concern to the MSC biology, the adipocytes, osteoblasts and chondrocytes, will be discussed in detail. The evidence for multi-potential progenitor cell populations from molecular and lineage analysis studies in vivo will be examined. Conceptual differences between mesenchymal stem cells and mesenchymal stromal cells and between mesenchymal stem cells and mesodermal stem cells will also be compared in the broader context of stem cell biology.

## Results and discussion

### Adipogenesis

Except for a small, cephalic neural crest-derived population in the head, all adipocytes in the adult body are of the mesoderm origin [32, 33]. Based on their morphology and location, adipocytes are categorized as either of a white or brown adipose tissue type (WAT and BAT, respectively) [34–38]. WAT adipocytes function as energy store and BAT adipocytes as heat dissipater. A third, minor type (brite or beige adipocytes) exhibits an intermediate feature with their location associated with WATs and their function resembling BAT adipocytes [39]. It is noteworthy that cellular and molecular features associated with mammalian adipocytes, e.g., regulated fusion of cytoplasmic lipid droplets through perilipins and respiratory uncoupling of lipid breakdown through mitochondrial uncoupling protein UCPs, are evolutionarily ancient and are present not only in adipocytes, but also in other mesoderm cell lineages and in cells derived from other germ layers [40–43]. The WAT adipocytes, of relevance to the MSC biology, are further divided into visceral and subcutaneous subtypes [34–38]. The visceral WATs have recently been shown to come from the splanchnic/visceral lateral plate mesoderm (LPM) [44]. The subcutaneous WATs, which constitute the bulk of human body fat and are found mainly in the abdominal and gluteofemoral regions, are primarily derived from the somatic/parietal LPM (discussed in more detail below). Understanding WAT ontogeny and adipogenesis in development therefore requires proper understanding of the LPM.

### Chondrogenesis and osteogenesis

These are two separate, but tightly-linked skeletogenic processes. Neural crest-derived cells make a significant contribution to the cranial bones and cartilages [45–47]. All other skeletal elements are of mesoderm origin [48, 49]. With the exception of the clavicle, which is generated through a mixture of intramembranous and endochondrial ossification [50], all post-cranial bones form through endochondrial ossification, i.e., secretion of bone-specific matrix proteins and subsequent mineralization of this matrix take place in a tissue architecture modeled by the chondrocytes. Therefore, percentage-wise, most cartilaginous tissues in the embryo exist only temporarily. Three mesoderm lineages in development, the axial, paraxial and lateral plate, are capable of generating skeletal elements. The axial mesoderm gives rise to the embryonic notochord and the adult nucleus pulposus and expresses many cartilage-specific markers (e.g., type II collagen and aggrecan) [51, 52]. However, cellular morphogenesis in the notochord and the nucleus pulposus is very different from what is known for the cartilage and these axial mesoderm-derived cells are generally not considered to be chondrocytes. The paraxial/somitic mesoderm generates all axial and associated skeletal elements (the vertebrae, ribs and part of the shoulder girdle), whereas all distal skeletal elements (bones in the limbs, the pelvic girdle, the sternum and part of the shoulder girdle) are derived from the somatic/parietal layer of the LPM [53, 54]. In endochondrial bones, chondrogenic differentiation of osteochondrogenic progenitors proceeds first, and osteogenic differentiation takes place later from a pool of quiescent progenitors located on the cartilage surface (perichondrium) [55]. Recent reports suggested that osteogenesis can also take place through chondrocyte de-differentiation or transdifferentiation [56, 57].

### Major lineages of the mesoderm germ layer

**Fig. 1:**
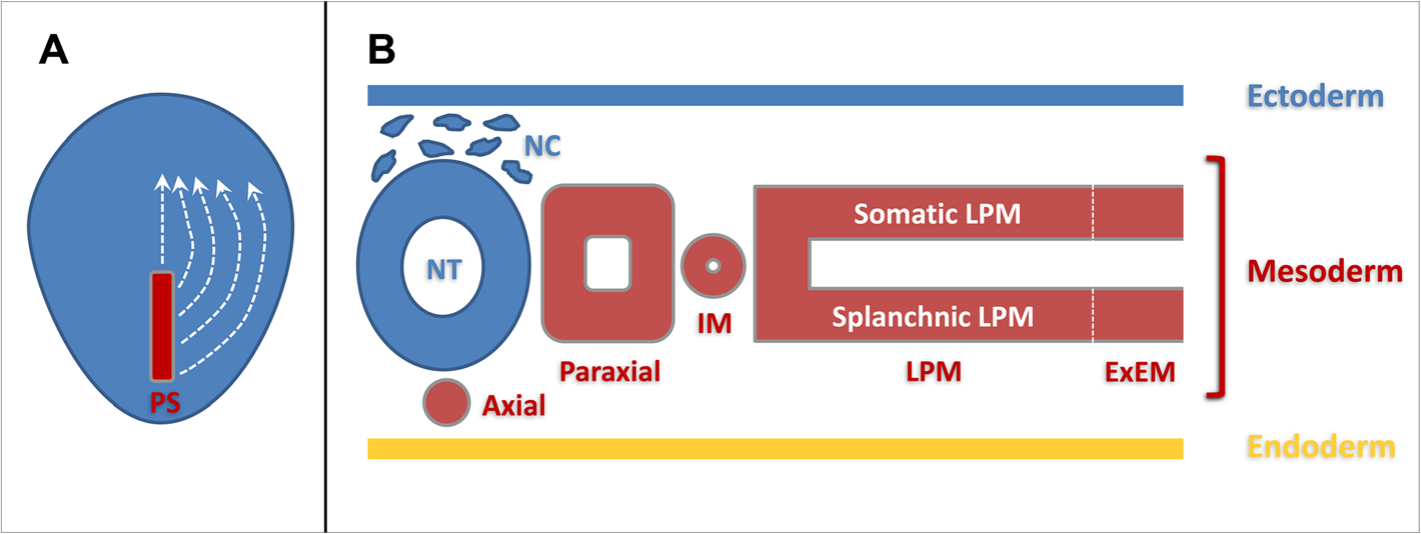
Schematic diagram of mesoderm formation and patterning during vertebrate early development. **a** Mesoderm precursors located in the primitive streak (PS) undergo epithelial-to-mesenchymal transition (EMT) and migrate between the ectoderm and endoderm germ layers to their final destinations in a spatially and temporally coordinated manner (white stippled lines). **b** Major mesoderm lineages (axial, paraxial, intermediate, lateral plate and extraembryonic) are laid out along the medio-lateral axis of the early embryo. IM: intermediate mesoderm; LPM: lateral plate mesoderm; ExEM: extraembryonic mesoderm; NT: neural tube; NC: neural crest. White stippled line represents the boundary between the LPM and ExEM

The above brief overview indicates that the three major cell types of special interest to the MSC biology share a common connection to the somatic LPM. Prior to the onset of gastrulation which generates an embryo with three germ layers, mesoderm precursors are specified molecularly when they are still part of the epiblast [58–61]. These mesoderm precursor cells ingress from the epiblast to become bona fide mesoderm cells through an epithelial to mesenchymal transition (EMT) process, which takes place in an embryonic structure called primitive streak [62–66]. EMT of mesoderm precursors at the primitive streak proceeds in a temporally and spatially ordered manner (Fig. 1a). Temporally speaking, early-ingressing precursor cells will migrate early and contribute to rostrally-located mesoderm populations. Those ingressing later from the primitive streak will form progressively more caudal mesoderm populations. The temporal difference in mesoderm EMT therefore translates into an antero-posterior difference in their final destination. Spatially speaking, those precursor cells that ingress from the anterior end of the primitive streak will contribute to the medially-located (embryologically dorsal) mesoderm lineages, e.g., the notochord and the medial half of the paraxial mesoderm. Those ingressing from the posterior end of the primitive streak will give rise to lateral (embryologically ventral) mesoderm lineages, e.g., the extraembryonic mesoderm (all mesoderm cells in the yolk sac, allantois, amnion and the fetal part of the chorio-allantoic placenta). Therefore, the antero-posterior spatial difference in mesoderm EMT translates into a medio-lateral (embryologically dorso-ventral) difference in their final destination. In such spatial coordinates, the LPM lineage lies between the intermediate mesoderm (giving rise to the urogenital tract) and the extraembryonic mesoderm, and is considered as the lateral-most (embryologically ventral-most) intraembryonic mesoderm lineage (Fig. 1b). The laterally-positioned LPM in a flat-disc shaped embryo is brought to its final ventral position during the process of body wall closure.

### The LPM and its somatic and splanchnic layers

**Fig. 2:**
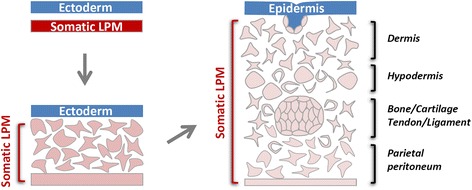
Schematic diagram of somatic LPM morphogenesis. Epithelial-to-mesenchymal transition (EMT) of the epithelial somatic LPM produces a homogenous mesenchymal cell population located between the ectoderm and the remaining LPM epithelium. These mesenchymal cells differentiate into many mesoderm lineageslineages, including the adipocytes, chondrocytes and osteoblasts. MSCs are hypothesized to exist both in the naïve somatic LPM population and in a more differentiated LPM tissue environment

Nascent mesoderm cells do not maintain their mesenchymal morphology for long. All mesoderm cells undergo at least one round MET (mesenchymal to epithelial transition) after their initial EMT, and many undergo several rounds of subsequent EMT/MET processes before their final differentiation. Nascent LPM cells polarize after a brief period of cell migration to form two epithelial layers, one located adjacent to the endoderm and called the splanchnic/visceral layer and the other located adjacent to the ectoderm and called the somatic/parietal layer (Fig. 1b). The apical side of both epithelial layers faces the enclosed internal space, the coelomic cavity. Cells from the splanchnic layer of the LPM contribute to nearly the entire cardiovascular system, including the cardiac and smooth muscles, endothelial cells, pericytes and HSCs, and to the mesothelial lining of visceral organs and visceral adipocytes. The somatic layer of the LPM gives rise to the dermis and hypodermis in the lateral and ventral body wall, the chondrocytes and osteocytes in all distal skeletal elements as discussed above, the vast majority of subcutaneous adipocytes including those in the abdominal and gluteofemoral regions and to potential resident progenitor and stem cells (MSC-like cells) in adipose and bone marrow tissues (Fig. 2). Ontologically speaking, therefore, attention concerning the embryonic origin and molecular regulation of MSCs in vivo should be focused on the epithelial-shaped somatic LPM and its subsequent EMT, proliferation, morphogenesis and lineage diversification. The splanchnic LPM, albeit vital for the body’s cardiovascular and hematopoietic functions, does not contain the full differentiation potential for mesoderm cell lineages attributed to the MSCs.

### In vivo evidence for multipotential progenitors in the somatic layer of the LPM

The ontological evidence discussed above suggests the three cell types of interest to the MSC biology have the somatic LPM as their common developmental origin. It is also evident that the majority of the bone and adipose tissues that are used as clinical sources for MSC enrichment/purification in vitro are derived from the somatic LPM. Although differentiation of MSCs in vitro may not necessarily mimic their in vivo behavior, it is worthwhile first reviewing the in vivo evidence for the existence of MSC-like progenitor/stem cell populations in the somatic LPM.

The somatic LPM, after its formation but before its differentiation, expresses many genes (e.g., αSMA) which are considered to be MSC markers later on [67–71]. Inducible αSMA-Cre-mediated lineage labeling suggested that αSMA positive cells in the bone marrow and periosteum are positive for MSC markers and these cells can give rise to chondrocytes and osteoblasts during fracture repair in vivo and to multiple mesenchymal lineages in vitro [72]. Furthermore, αSMA also marks an adult adipose stem cell population [73] which resembles adipose and bone marrow MSCs with multilineage differentiation potential in vitro [70, 71]. TWIST1, another marker for early somatic LPM [74], is highly expressed together with its close homolog TWIST2 in primary MSCs [75, 76]. TWIST1 and TWIST2 expression prevents MSC differentiation in vivo [75, 76] and TWIST2-positive cells were shown to give rise to both osteoblasts and chondrocytes in vivo in a Cre-mediated lineage analysis [77].

The epithelial-shaped early somatic LPM cells differentiate by undergoing EMT and generate a population of mesenchymal cells located between the overlying ectoderm and the remaining epithelial somatic LPM (the future parietal mesothelium). This population of somatic LPM-derived mesenchymal cells is present throughout the antero-posterior axis of the embryo, but is most prominent in areas where limb buds develop [78]. These mesenchymal cells are initially homogenous and at the limb levels they are uniformly positive for Prx1, a paired-related homeobox gene [79]. Results from virus-mediated single-cell labeling of the limb mesenchyme suggest that these are multi-potential cells capable of giving rise to two or more of the limb cell types, including those in the cartilage, perichondrium, tendon, muscle connective and dermis [80]. Sox9, an early molecular marker for chondrogenic condensation, labels in fact a multi-lineage sub-population of these mesenchymal cells, including those contributing to the cartilage, bone, tendon and ligament [81, 82]. Regulated by a pre-adipocyte marker Pref-1, Sox9 also inhibits the adipogenic potential of these mesenchymal cells [83].

In a more differentiated tissue environment derived from the somatic LPM, multi-potential progenitors have been shown to exist for the osteoblasts, adipocytes and perivascular stromal cells by Osterix-Cre mediated lineage labeling [84], for the osteoblasts, chondrocytes, endothelial and bone marrow stromal cells by Nestin-Cre mediated lineage analysis [27, 85], for the osteoblasts, chondrocytes and marrow stromal cells by Gremlin1-Cre mediated lineage and Rainbow Actin-Cre mediated clonal analyses [86, 87], for the chondrocytes and osteoblasts by Col2-Cre mediated lineage labeling [88], and for the osteoblasts and adipocytes by fate-mapping and transplantation analyses of leptinR+ cells in the adult bone marrow [89].

### Mesenchymal stem cells or mesenchymal stromal cells

Taken together, data from the ontological, molecular and cellular analyses strongly support the hypothesis that there exists a MSC-like progenitor population both during somatic LPM differentiation and during homeostatic maintenance of somatic LPM-derived tissues. The multi-potential stemness of MSCs in vivo, with the capacity to generate tissues including the bone, cartilage, tendon, muscle, fat and marrow stroma [8], however, has not been satisfactorily demonstrated. This gap can be viewed from three perspectives in the broader context of stem cell biology.

**Fig. 3:**
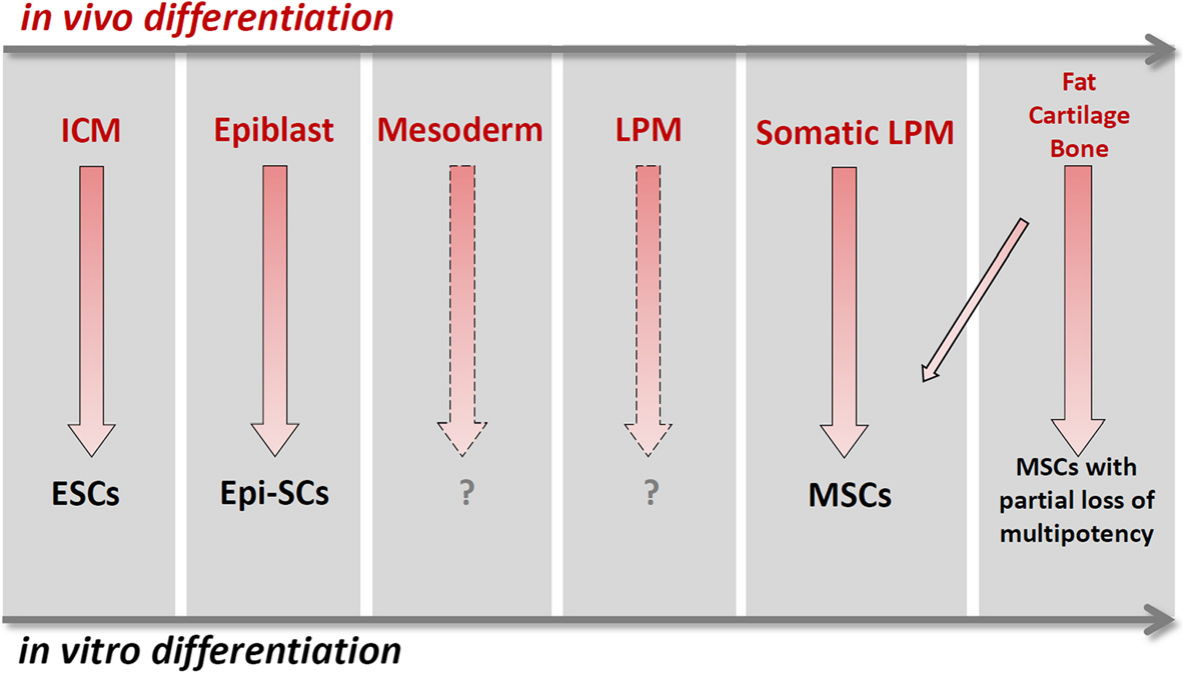
Comparison of MSC-related phenomena in vivo and in vitro. In vivo, progenitor populations that will give rise to the adipocyte, chondrocyte and osteoblast lineages pass through developmental phases that progressively restrict their fate choices. This is correlated with progressively limited differentiation potentials of corresponding stem cell populations cultured in vitro. ICM: inner cell mass; ESCs: embryonic stem cells; Epi-SCs: epiblast stem cells; LPM: latral plate mesoderm; MSCs: mesenchymal stem cells. Corresponding stem cell populations for the mesoderm (pan-mesoderm stem cells) and LPM (pan-lateral plate mesoderm stem cells) have not yet been reported

First, the nature of MSC multipotency in vivo may require further clarification and more precise definition (Fig. 3). Uni-potential stem-like behavior of MSC-related lineages has been reported in many studies, and bi-potential or multi-potential stemness of closed related lineages (such as the chondrocytes, osteoblasts and bone marrow stromal cells) has also been documented [4, 27, 86, 87, 90]. However, multi-potential stemness with differentiation potentials for all MSC-related cell lineages has not been demonstrated yet. An evolving concept of the MSCs, similar to that of the HSCs, is to view them as a heterogeneous mix of sub-populations each harboring a unique set of multipotency [3, 4, 86, 87, 91]. Such a concept is in agreement with the findings that some MSCs may represent a subpopulation of pericytes or fibroblasts in vivo [92–95]. This modified concept of MSCs, however, does not preclude the potential existence of a bona fide multi-potential mesenchymal stem cell population either in development or during adult tissue homeostasis. Secondly, many well accepted concepts, such as the embryonic stem cells (ESCs) and epiblast stem cells (Epi-SCs), represent in vitro phenomena without embryological counterparts. Although ESCs have been likened to the inner cell mass (ICM) during early mammalian development and Epi-SCs to the young epiblast before gastrulation (Fig. 3), neither the ICM nor the epiblast in vivo meets the stemness criteria either from a single cell or from a cell population point of view. These cells, like the MSCs, are multi-potential progenitor cells in the so-called “epigenetic landscape” of successive lineage restrictions during animal development [96]. Nevertheless, the stem cell nature or the in vivo relevance of ESCs and Epi-SCs has seldom been questioned in the scientific literature, and likewise the MSCs should not either. Thirdly, the ESCs and EpiSCs are derived from embryologically-speaking more naïve tissues. As a consequence, cellular purity and molecular homogeneity of ESCs and Epi-SCs are much more rigorously defined and regulated than the MSCs. Better characterization and standardization of MSC sources in vivo and cellular and molecular features in vitro is therefore essential for the future progress of MSC biology. Promising novel sources for MSC derivation are the naïve somatic LPM, the undifferentiated population of somatic LPM-derived mesenchyme and the somatic LPM-like cell populations derived from guided differentiation of ESCs, Epi-SCs or iPSCs.

### Mesenchymal stem cells or mesodermal stem cells

Although most of the cell and tissue types which have been associated with MSCs come ontologically from the somatic LPM, many of them do not. For example, as target differentiation lineages, the cardiac and most of the smooth muscles come from the splanchnic LPM and the skeletal muscles are from the somitic mesoderm. As tissue sources, the neural crest is of ectoderm origin, and axial bones and dorsal dermis are of somitic mesoderm origin. The peripheral blood in the adult and the umbilical cord blood in the fetus are of splanchnic LPM/splanchnic extraembryonic mesoderm origin, the amniotic mesoderm is of somatic extraembryonic mesoderm origin and the placental and the umbilical cord mesenchyme is derived from a mixture of somatic and splanchnic extraembryonic mesoderm cells.

Leaving aside the neural crest-derived cell populations (not discussed here), one may ask whether the term “mesodermal stem cells” is more suitable to describe the properties associated with the MSCs. To answer this question one needs to take a fresh look at the epigenetic landscape proposed by Waddington about 60 years ago [96]. The original concept of epigenetic landscape of animal development outlined progressive restriction of differentiation potentials from a totipotent zygote to terminally differentiated functional cell types. Uni-, bi- or multi-potential stem cells are perceived to exist in each branch or at the branching point. Cell lineages that are closely related ontologically are more likely to be represented by a common progenitor/stem cell population and those distantly related less likely so. For example, paths and barriers for the germ cells and the soma and for the three principal germ layers are set early in development and fate conversion crossing these barriers is almost never observed in vivo. One may therefore argue that the MSCs are a sub-population of, and are distinct from, a pan-mesodermal stem cell population, the latter of which can be viewed as the equivalent of primitive streak-like mesoderm progenitors in vivo (Fig. 3). Although other sub-populations of lineage-biased, multi-potential mesodermal stem cells may exist, cell types not strictly derived with the somatic LPM should preferably not be associated with the MSCs.

Advances from iPSC-related research, however, have complicated such a simplistic paradigm. Reversion from a terminally differentiated fate to a pluripotent fate can now be achieved relatively easily through molecular or chemical perturbations of this epigenetic landscape and paths of reprogramming may not necessarily mirror the conventional landscape of progressive differentiation in vivo. As a consequence, it is unclear whether the knowledge obtained from in vivo studies should serve as the guiding principle for achieving fate reversion and targeted lineage differentiation in vitro. It will be fascinating to see whether the epigenetic landscape manifested in vitro can teach us about unrealized potentials in vivo. MSC studies in vitro therefore should not be confined by MSC behaviors in vivo.

## Conclusions

This article has outlined the ontological, molecular and cellular evidence in support of the existence of MSCs in vivo. The somatic LPM is the most important mesoderm compartment for the cells and tissues commonly associated with MSCs. The term “mesenchymal stem cells” is preferred by this author and a clear distinction should be made between somatic LPM-derived MSCs and mesenchymal-shaped stem-like cells derived from other mesodermal compartments. Such understanding of MSCs based on in vivo evidence would benefit in vitro endeavors in harnessing the therapeutic powers of MSCs. The ultimate goal of MSC-related research is to integrate in vitro-reconstituted cells or tissues into an in vivo environment, which will be facilitated by an awareness of how endogenous MSC populations differentiate and self-organize in vivo.

## Methods

Views expressed in this article are based partially on research carried out in the author's laboratory at RIKEN Center for Developmental Biology. All animal experiments performed in the author's laboratory were approved by the institute's ethical committee.
